# Impact of Tissue Damage and Hemodynamics on Restenosis Following Percutaneous Transluminal Angioplasty: A Patient-Specific Multiscale Model

**DOI:** 10.1007/s10439-024-03520-1

**Published:** 2024-05-03

**Authors:** Anna Corti, Matilde Marradi, Cemre Çelikbudak Orhon, Francesca Boccafoschi, Philippe Büchler, Jose F. Rodriguez Matas, Claudio Chiastra

**Affiliations:** 1 Department of Electronics, Information and Bioengineering, Politecnico di Milano, Via Ponzio 34/5, 20133 Milan, Italy; 2 Laboratory of Biological Structure Mechanics (LaBS), Department of Chemistry, Materials and Chemical Engineering “Giulio Natta”, Politecnico di Milano, Milan, Italy; 3 Department of Cell Biology-Inspired Tissue Engineering, MERLN Institute for Technology-Inspired Regenerative Medicine, Maastricht University, Maastricht, The Netherlands; 4 Laboratory of Hemodynamics and Cardiovascular Technology, Institute of Bioengineering, Ecole Polytechnique Fédérale de Lausanne, Lausanne, Switzerland; 5 Department of Health Sciences, University of Piemonte Orientale “A. Avogadro”, Novara, Italy; 6 ARTORG Center for Biomedical Engineering Research, University of Bern, Bern, Switzerland; 7 PolitoBIOMed Lab, Department of Mechanical and Aerospace Engineering, Politecnico di Torino, Turin, Italy

**Keywords:** Peripheral artery disease (PAD), Arterial wall remodeling, Mechanobiology, Agent-based modeling (ABM), Finite element analysis (FEA), Computational fluid dynamics (CFD)

## Abstract

Multiscale agent-based modeling frameworks have recently emerged as promising mechanobiological models to capture the interplay between biomechanical forces, cellular behavior, and molecular pathways underlying restenosis following percutaneous transluminal angioplasty (PTA). However, their applications are mainly limited to idealized scenarios. Herein, a multiscale agent-based modeling framework for investigating restenosis following PTA in a patient-specific superficial femoral artery (SFA) is proposed. The framework replicates the 2-month arterial wall remodeling in response to the PTA-induced injury and altered hemodynamics, by combining three modules: (i) the PTA module, consisting in a finite element structural mechanics simulation of PTA, featuring anisotropic hyperelastic material models coupled with a damage formulation for fibrous soft tissue and the element deletion strategy, providing the arterial wall damage and post-intervention configuration, (ii) the hemodynamics module, quantifying the post-intervention hemodynamics through computational fluid dynamics simulations, and (iii) the tissue remodeling module, based on an agent-based model of cellular dynamics. Two scenarios were explored, considering balloon expansion diameters of 5.2 and 6.2 mm. The framework captured PTA-induced arterial tissue lacerations and the post-PTA arterial wall remodeling. This remodeling process involved rapid cellular migration to the PTA-damaged regions, exacerbated cell proliferation and extracellular matrix production, resulting in lumen area reduction up to 1-month follow-up. After this initial reduction, the growth stabilized, due to the resolution of the inflammatory state and changes in hemodynamics. The similarity of the obtained results to clinical observations in treated SFAs suggests the potential of the framework for capturing patient-specific mechanobiological events occurring after PTA intervention.

**Supplementary Information:**

The online version contains supplementary material available at 10.1007/s10439-024-03520-1.

## Introduction

Peripheral artery disease (PAD), which affects more than 230 million adults worldwide, is the third leading cause of atherosclerosis-related morbidity [1–3]. It often manifests in the superficial femoral artery (SFA) [1–3]. Percutaneous transluminal angioplasty (PTA) stands out as one of the most widely employed endovascular procedures for treating PAD in the SFA. However, the insurgence of restenosis, resulting in the re-narrowing of the treated vessel, represents a major adverse event, ultimately leading to a suboptimal long-term outcome of the procedure (~60% 1-year primary patency rate) [4]. Restenosis occurs as a consequence of maladaptive healing processes in response to the severe vascular injury and endothelial denudation caused by PTA. These processes culminate into abnormal intimal growth due to inflammatory-driven, sustained synthetic, and proliferative activities of smooth muscle cells (SMCs) [5–7]. While the injury induced by the PTA procedure has been recognized as the primary trigger for inflammatory-driven vascular cell activation, post-intervention hemodynamics also appears to play a crucial role in the development and progression of restenosis, through pro-inflammatory mechanisms and both direct and endothelial-mediated effects on SMC activity [8–10]. To date, extensive research has been carried out with the aim of deciphering the underlying patient, biological, biomechanical, and operator-related factors and events contributing to the complex, multifactorial, and multiscale restenosis process [11–13]. However, a comprehensive understanding of the mechanobiological mechanisms underlying the pathogenesis of restenosis remains elusive.

Recently, computational multiscale agent-based modeling frameworks, integrating both continuum models and agent-based models (ABMs), have shown promising results in describing the relevant mechanobiological mechanisms underlying atherosclerosis and vascular adaptation processes following endovascular procedures, including restenosis after balloon angioplasty and/or stenting [11]. In the context of restenosis, previous models have mainly focused on factors related to either wall damage or hemodynamics [14–27]. Notably, Corti et al. [28] have proposed a multiscale agent-based modeling framework for simulating post-PTA arterial wall remodeling due to both PTA-induced wall damage and altered hemodynamics. This framework combines a finite element simulation of the PTA procedure, computational fluid dynamics (CFD) simulations, and an ABM of arterial wall remodeling. As a pilot study, the framework has been applied to an idealized model of diseased SFA, utilizing simplified characteristics for the arterial wall, namely isotropic hyperelastic material models coupled to a ductile damage model (as regards the intima material in the diseased portion) to simulate the progressive softening of the material under stretch and compute the damage induced during the expansion procedure. The present work builds upon the previous investigation [28] and aims to develop a patient-specific computational framework to investigate the combined influence of arterial wall damage and altered fluid dynamics in the development of restenosis in SFAs subsequent to PTA. This study introduces substantial enhancements compared to the previous investigation, encompassing (i) the application of the framework to a patient-specific SFA model, (ii) an improved mechanical description using a finite element model of the PTA procedure that takes into account the anisotropic hyperelastic material response of the tissue, coupled with a damage formulation for fibrous soft tissue [29], material properties calibrated on experimental data collected on fresh human SFA specimens, and an element deletion strategy to simulate injury-induced dissections and lacerations, and (iii) the implementation of SMC migration within the ABM. The framework was applied to a patient-specific SFA model in which the balloon was expanded to two different diameters to detect differences in the arterial wall response after PTA procedures.

## Materials and Methods

**Fig. 1:**
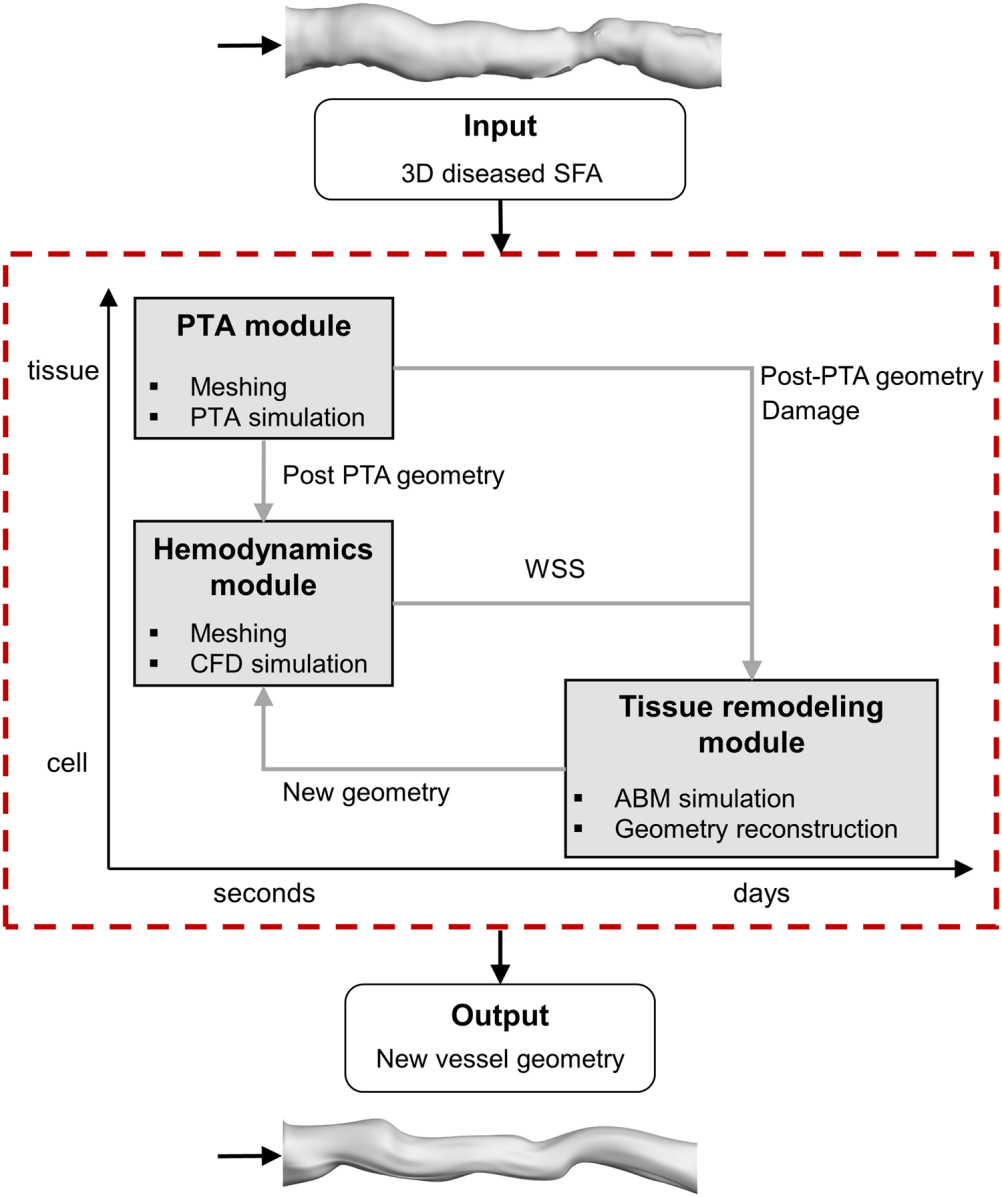
Multiscale computational framework. Starting from the diseased superficial femoral artery (SFA) model, the framework (dashed red box) simulates (i) the percutaneous transluminal angioplasty (PTA) procedure (PTA module) at the tissue-seconds scale, through a structural mechanics simulation, (ii) the post-intervention hemodynamics (hemodynamics module) at the tissue-second scale, through computational fluid dynamics (CFD) simulations, and (iii) the post-intervention arterial wall remodeling along 2 simulated months (tissue remodeling module) at the cell-days scale, through a bidimensional (2D) agent-based model (ABM). Finally, from the ABM outputs, a new three-dimensional (3D) vessel geometry is reconstructed

Figure 1 shows the framework of restenosis following PTA, as developed in a previous study by our research group [28] and herein applied to a patient-specific case. Briefly, the framework receives as input the diseased patient-specific SFA geometry (i.e., pre-PTA condition) and generates as output the remodeled vessel geometry at 2-month of follow-up. The framework (i) simulates the PTA procedure through a finite element structural mechanics simulation (PTA module), enabling the computation of the post-intervention arterial configuration and the intervention-induced arterial wall damage, (ii) computes the post-intervention hemodynamics through CFD simulations (hemodynamics module) and (iii) simulates the post-intervention arterial wall remodeling in response to the intervention-induced damage and hemodynamic stimuli through an ABM, generating the remodeled vessel geometry at prescribed follow-ups (tissue remodeling module). The hemodynamics—tissue remodeling module sequence was repeated at a predefined coupling period (1 month in the present study) to account for the hemodynamic change in the remodeled vessel geometry.

The framework was used to investigate the 2-month restenosis following PTA in one patient-specific SFA under two different intervention conditions, namely up to a balloon expansion diameter of 5.2 mm (case A) and 6.2 mm (case B). Since the patient’s artery had been originally treated with a 5 mm balloon, the simulated expansion of case A aimed to replicate the real PTA procedure, while case B represented an extreme scenario, with the purpose of demonstrating the effects of greater expansion (1 mm more on the diameter) on arterial wall remodeling following intervention.

### PTA module

**Fig. 2:**
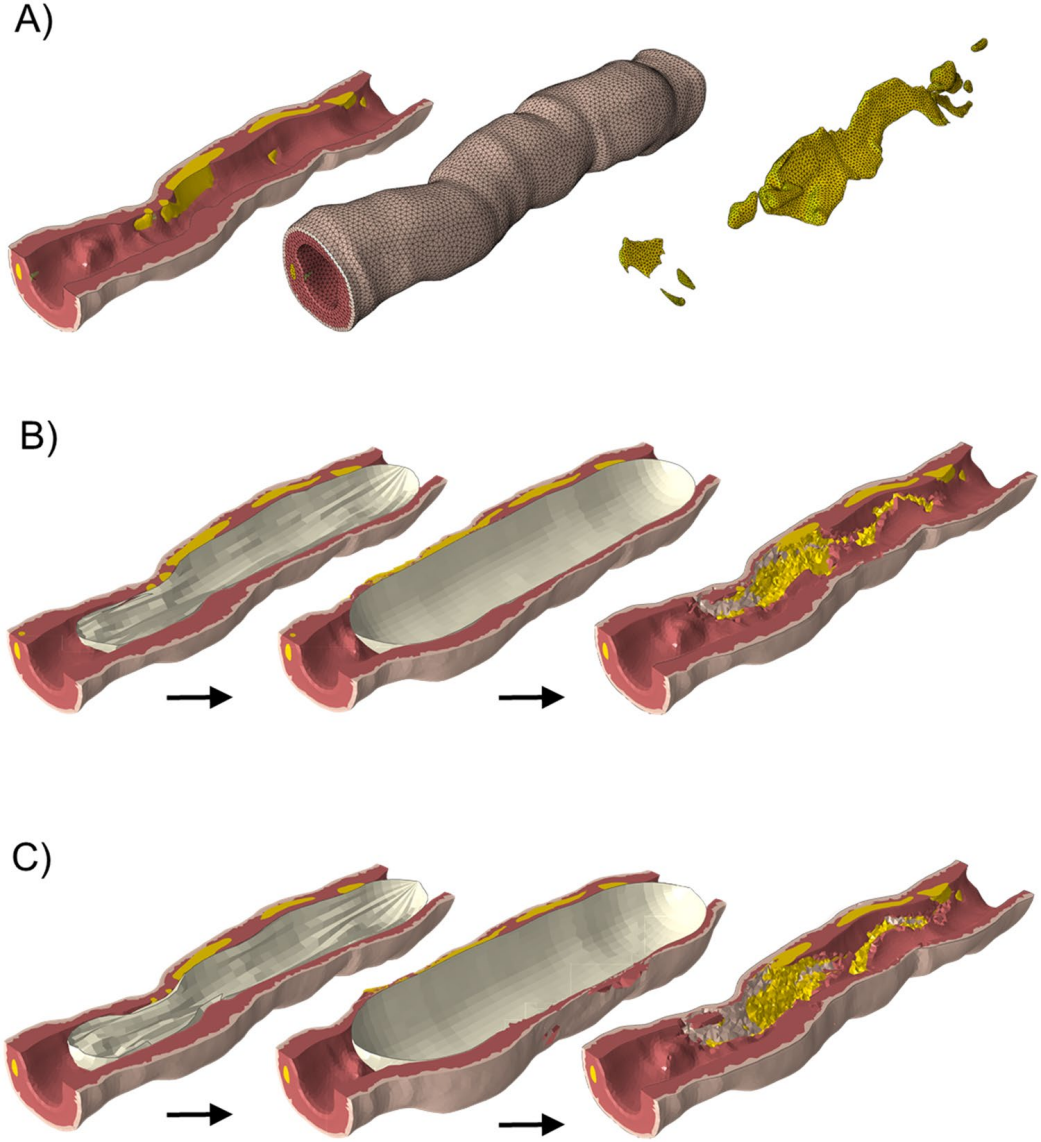
**A** Left: Three-dimensional superficial femoral artery model reconstructed from optical coherence tomography, composed by media and adventitia layers, and the atherosclerotic plaque (in yellow); Center: Tetrahedral mesh of the arterial wall; Right: tetrahedral mesh of the plaque. **B** Balloon expansion procedure for the case A. Left: half time of the expansion step; Center: end of the expansion step; Right: final geometry at the end of the percutaneous transluminal angioplasty procedure. **C** Balloon expansion procedure for the case B. Left: half time of the expansion step; Center: end of the expansion step; Right: final geometry at the end of the percutaneous transluminal angioplasty procedure

The patient-specific SFA geometrical model was reconstructed from optical coherence tomography (OCT) images at the University of Bern (Bern, Switzerland) by using Amira v5.4.5 (Thermo Fisher Scientific, Waltham, MA, USA), as extensively detailed in [30]. The patient imaging data belong to a study [30], approved by the ethical review committee of the Ethikkommission Nordwest- und Zentralschweiz (EKNZ 2014-119). In the study [30], all procedures were performed in accordance with the Declaration of Helsinki and informed consent was obtained from all individual participants. The SFA model presented length of 24 mm and inlet lumen diameter of 3.3 mm, inlet external diameter of 4.7 mm, outlet lumen diameter of 2.9 mm, and outlet external diameter of 5.0 mm (expressed as hydraulic diameters) (Fig. 2A). The arterial model was composed by two layers, namely the media layer (assumed to embed also the intima layer) and the adventitia layer, along with the atherosclerotic plaque. The arterial wall was discretized using 216,570 linear tetrahedral elements with one integration point (C3D4 elements) (Fig. 2A). The media, adventitia, and plaque tissues were modeled as hyperelastic materials with density of 1000 kg/m^3^ and assumed as quasi-incompressible (Poisson’s ratio of 0.485). Specifically, the mechanical behavior of the media and adventitia layers were described using the anisotropic hyperelastic constitutive model developed by Holzapfel et al. [31], which involves an isotropic matrix and two families of fibers oriented along two preferred directions, one coincident with the circumferential direction and one with the longitudinal direction. Moreover, to simulate damage mechanisms occurring in the media layer during PTA procedure, a damage model designed for fibrous soft tissues [29] was implemented, with strain energy function detailed in Table 1. The material parameters, provided in Table 2, were calibrated from experimental data on human distal SFA specimens. Additional information about the SFA specimens and the calibration process can be found in the Supplementary Material. The mechanical behavior of the plaque was described through an isotropic elastic-plastic material model. Specifically, the Neo-Hookean constitutive model was employed for the elastic part, while perfect plasticity was defined by setting the yield stress ${{\varvec{\sigma}}}_{{\varvec{y}}}$ and plaque strain ${{\varvec{\lambda}}}_{{\varvec{y}}}$. Furthermore, to simulate the damage mechanisms occurring within the plaque during balloon expansion, the ductile damage model available in Abaqus (Dassault Systèmes Simulia Corp., Johnston, RI, USA) was utilized by specifying the equivalent plastic strain at the initiation of damage ${{\varvec{u}}}_{{\varvec{f}}}^{{\varvec{p}}{\varvec{l}}}$ , and an exponential damage evolution with specified total dissipated energy per unit area ${{\varvec{G}}}_{{\varvec{f}}}$. Table 3 details the plaque material parameters corresponding to a moderate calcified plaque [32].

**Table 1:** Constitutive model of the media layer

SEF of the media layer	$\overline{W }\left(\overline{{\varvec{C}} },{{\varvec{A}}}_{1},{{\varvec{A}}}_{2}\right)=\left(1-{d}^{m}\right){\overline{W} }_{0}^{m}\left(\overline{{\varvec{C}} }\right)+\left(1-{d}_{{A}_{1}}^{f}\right){\overline{W} }_{0}^{f}\left(\overline{{\varvec{C}} },{{\varvec{A}}}_{1}\right)+\left(1-{d}_{{A}_{2}}^{f}\right){\overline{W} }_{0}^{f}\left(\overline{{\varvec{C}} },{{\varvec{A}}}_{2}\right)$
Isochoric SEF of undamaged matrix	${\overline{W} }^{m}\left({\overline{I} }_{1}\right)={c}_{10}\left({\overline{I} }_{1}-3\right)$ ${\overline{I} }_{1}=tr\overline{{\varvec{C}} }$
Isochoric SEF of undamaged fibers	${\overline{W} }^{f}\left({\overline{I} }_{4},{\overline{I} }_{6}\right)=\frac{{k}_{1}}{{2k}_{2}}\left({e}^{{k}_{2}{\langle {\overline{E} }_{4}\rangle }^{2}}-1\right)+\frac{{k}_{3}}{{2k}_{4}}\left({e}^{{k}_{4}{\langle {\overline{E} }_{6}\rangle }^{2}}-1\right)$ $$\begin{array}{c}{\overline{E} }_{4}=\kappa \left({\overline{I} }_{1}-3\right)+\left(1-3\kappa \right)\left({\overline{I} }_{4}-1\right)\end{array}$$ $$\begin{array}{c}{\overline{E} }_{6}=\kappa \left({\overline{I} }_{1}-3\right)+\left(1-3\kappa \right)\left({\overline{I} }_{6}-1\right)\end{array}$$ $$\begin{array}{c}\langle {\overline{E} }_{4}\rangle =\left\{\begin{array}{c}{\overline{E} }_{4} \,\,if \,{\overline{E} }_{4}\ge 0\\ 0 \,otherwise\end{array}\right. \langle {\overline{E} }_{6}\rangle =\left\{\begin{array}{c}{\overline{E} }_{6} \,if\, {\overline{E} }_{6}\ge 0\\ 0 \,otherwise\end{array}\right.\end{array}$$ ${\overline{I} }_{4}=\overline{{\varvec{C}} } : {{\varvec{A}}}_{1}$ ${\overline{I} }_{6}=\overline{{\varvec{C}} } :{{\varvec{A}}}_{2}$ ${\varvec{A}}_{1} = {\varvec{n}}_{c} \otimes {\varvec{n}}_{c} \quad{\varvec{A}}_{2} = {\varvec{n}}_{l} \otimes {\varvec{n}}_{l}$
Damage mechanisms of the matrix	$$\begin{array}{c}{d}^{m}\left(\overline{{\varvec{C}} }\right)={\left({s}^{m}\left(\overline{{\varvec{C}} }\right)\right)}^{3}\cdot \left[10-15\cdot {s}^{m}\left(\overline{{\varvec{C}} }\right)+6\cdot {{(s}^{m}(\overline{{\varvec{C}} }))}^{2}\right]\end{array}$$ ${s}^{m}\left(\overline{{\varvec{C}} }\right)=\frac{{\Xi }^{m}\left(\overline{{\varvec{C}} }\right)-{\Xi }_{0}^{m}}{{\Xi }_{F}^{m}-{\Xi }_{0}^{m}}$ $$\begin{array}{c}{\Xi }^{m}\left(\overline{{\varvec{C}} }\right)=\sqrt{2{\overline{W} }_{0}^{m}\left(\overline{{\varvec{C}} }\right)}\end{array}$$
Damage mechanisms of the fibers	$$\begin{array}{c}{s}_{{A}_{n}}^{f}\left(\overline{{\varvec{C}} },{{\varvec{A}}}_{n}\right)=\frac{{\Xi }^{f{A}_{n}}\left(\overline{{\varvec{C}} },{{\varvec{A}}}_{n}\right)-{\Xi }_{0}^{f{A}_{n}}}{{\Xi }_{F}^{f{A}_{n}}-{\Xi }_{0}^{f{A}_{n}}}\end{array}$$ $$\begin{array}{c}{d}_{{A}_{n}}^{f}\left(\overline{{\varvec{C}} },{{\varvec{A}}}_{n}\right)={\left({s}_{{A}_{n}}^{f}\left(\overline{{\varvec{C}} },{{\varvec{A}}}_{n}\right)\right)}^{3}\cdot \left[10-15\cdot {s}_{{A}_{n}}^{f}\left(\overline{{\varvec{C}} },{{\varvec{A}}}_{n}\right)+6\cdot {\left({s}_{{A}_{n}}^{f}\left(\overline{{\varvec{C}} },{{\varvec{A}}}_{n}\right)\right)}^{2}\right]\end{array}$$ ${\Xi }^{f{A}_{n}}\left(\overline{{\varvec{C}} },{{\varvec{A}}}_{n}\right)=\sqrt{2{\overline{W} }_{0}^{f}\left(\overline{{\varvec{C}} },{{\varvec{A}}}_{n}\right)}$

*SEF* strain energy function; ${\overline{W} }_{0}^{m}$ isochoric SEF of undamaged matrix; ${\overline{W} }_{0}^{f}$ isochoric SEF of undamaged fibers; ${d}^{m}$ damage variable of matrix; ${d}_{{A}_{1}}^{f}$ and ${d}_{{A}_{2}}^{f}$ damage variable of fibers; $\overline{{\varvec{C}} }$ is the isochoric contribution of the right Cauchy-Green strain tensor; ${\varvec{A}}$ is the second order tensor characterizing fibrous architecture of the artery wall, with ${{\varvec{n}}}_{{\varvec{c}}}$ and ${{\varvec{n}}}_{{\varvec{l}}}$ unit vectors in the circumferential and longitudinal directions of the artery respectively; ${\overline{I} }_{1}$, ${\overline{I} }_{4}$ and ${\overline{I} }_{6}$: invariants of $\overline{{\varvec{C}} }$; ${c}_{10}$, ${k}_{1}$, ${k}_{2}$, ${k}_{3}$, ${k}_{4}$: strain energy function parameters, detailed in Table 2; ${\Xi }_{0}$, ${\Xi }_{F}$: energy-like parameters representing the initiation and end of the damage evolution, detailed in Table 2

**Table 2:** Arterial tissue parameters

Parameter	Media	Adventitia
${{\varvec{c}}}_{10}$[MPa]	0.183	0.104
${{\varvec{k}}}_{1}$[MPa]	0.080	0.168
${{\varvec{k}}}_{2}$[-]	0.253	0.009
${{\varvec{k}}}_{3}$[MPa]	0.127	0.205
${{\varvec{k}}}_{4}$[-]	0.217	0.009
${\boldsymbol{\Xi }}_{0}^{{\varvec{m}}}$[MPa^-1/2^]	0.497	–
${\boldsymbol{\Xi }}_{{\varvec{F}}}^{{\varvec{m}}}$[MPa^-1/2^]	0.808	–
${\boldsymbol{\Xi }}_{0}^{{\varvec{f}}{{\varvec{A}}}_{1}}$[MPa^-1/2^]	0.005	–
${\boldsymbol{\Xi }}_{{\varvec{F}}}^{{\varvec{f}}{{\varvec{A}}}_{1}}$[MPa^-1/2^]	2.323	–
${\boldsymbol{\Xi }}_{0}^{{\varvec{f}}{{\varvec{A}}}_{2}}$[MPa^-1/2^]	0.040	–
${\boldsymbol{\Xi }}_{{\varvec{F}}}^{{\varvec{f}}{{\varvec{A}}}_{2}}$[MPa^-1/2^]	2.179	–

${{\varvec{c}}}_{10}$, ${{\varvec{k}}}_{1}$, ${{\varvec{k}}}_{2}$, ${{\varvec{k}}}_{3}$, ${{\varvec{k}}}_{4}$: strain energy function parameters; ${\boldsymbol{\Xi }}_{0}$, ${\boldsymbol{\Xi }}_{{\varvec{F}}}$: energy-like parameters representing the initiation and end of the damage evolution

**Table 3:** Plaque material parameters

**Parameter**	**Value**
${{\varvec{c}}}_{10}$[MPa]	0.0863
${{\varvec{\sigma}}}_{{\varvec{y}}}$[MPa]	0.43
${{\varvec{\lambda}}}_{{\varvec{y}}}$[-]	1.75
${{\varvec{G}}}_{{\varvec{f}}}$[*mJ*/${{\varvec{m}}{\varvec{m}}}^{2}$]	0.0215
${{\varvec{u}}}_{{\varvec{f}}}^{{\varvec{p}}{\varvec{l}}}$[-]	0.1

${{\varvec{\sigma}}}_{{\varvec{y}}}$: Cauchy yield stress; ${{\varvec{\lambda}}}_{{\varvec{y}}}$: yield stretch; ${{\varvec{G}}}_{{\varvec{f}}}$: fracture energy; ${{\varvec{u}}}_{{\varvec{f}}}^{{\varvec{p}}{\varvec{l}}}$: equivalent plastic strain at failure

The Armada 35 PTA balloon (Abbott Laboratories, Abbott Park, IL, USA) was considered for the PTA simulation. Two balloon models were created in SolidWorks (Dassault Systèmes, SolidWorks Corp., Waltham, MA, USA) in their crimped configuration, with a multi-wing structure [33–35], and presented a length of 19.0 mm and a nominal diameter of 5.00 mm (balloon A) and 6.00 mm (balloon B). The balloon thickness was set to 0.025 mm [34]. The polymeric material of the balloon was modeled through a linear elastic isotropic model with Poisson’s ratio of 0.3 and Young’s modulus of 1.45 GPa, and material density was set to 1256 kg/m^3^ [36].

 Before performing the PTA simulations, the zero-pressure artery configuration was determined using a pull-back algorithm [37], implemented in Matlab (MathWorks, Natick, MA, USA). The zero-pressure artery configuration corresponds to the reference configuration that, when loaded with the diastolic pressure, deforms into the artery configuration reconstructed from OCT images. The algorithm iteratively updates the nodal coordinates of the zero-pressure configuration until the absolute maximum nodal distance between the OCT-reconstructed artery configuration and the loaded zero-pressure configuration is lower than a tolerance, set as 0.001 mm [37]. The PTA procedure was simulated using the finite element solver Abaqus/Explicit (Dassault Systèmes Simulia Corp., Johnston, RI, USA) and by considering the element deletion method, according to which medial and plaque elements reaching a damage value of 0.8 were removed from the mesh throughout the simulation. The deletion of failed elements enabled the replication of arterial wall tearing and dissections due to balloon expansion, besides avoiding numerical difficulties arising from excessive element distortion. The PTA simulations consisted of the following steps: (i) pressurization of the SFA model, through the application of a uniform pressure of 120 mmHg to the internal surface of the vessel (this load was sustained throughout the entire simulation), (ii) balloon inflation, by applying a smooth loading pressure to the internal surface of the balloon of 12 and 9 atm for balloon A and B, respectively, and establishing the interaction between the balloon and the artery wall, (iii) balloon deflation, by removing the load applied to the internal surface of the balloon, and (iv) stabilization, by deactivating the interaction between the balloon and the artery wall to mimic balloon removal. Each of these procedural steps was simulated as a quasi-static process, by ensuring that the ratio between kinetic and internal energy was below 5% during the entire simulation [34]. The general contact algorithm was employed to establish the interaction between the balloon and the arterial wall. The interaction was characterized by a ‘hard’ normal behavior and a tangential behavior with a friction coefficient of 0.2 [34]. Both exterior and interior surfaces of the arterial wall elements were designated as interaction surfaces to consider potential contacts that might occur due to element deletion. Regarding the boundary conditions, the nodes of the vessel and balloon extremities were constrained from motion, with the exception of the distal tip of the balloon, where only axial translation was permitted. At the end of the PTA simulation, the post-PTA vessel geometry was reconstructed in Matlab by applying the nodal displacements to the initial nodal coordinates. Figure 2 (panels B and C) shows the balloon expansion procedure (half and full inflation), as well as the final and processed vessel geometries obtained after the PTA procedure, for cases A and B. The lumen surface of the post-PTA geometry was considered for the CFD simulation (Sect. “Hemodynamics module”) while the nodal coordinates of the entire geometry together with the associated damage variable were used to initialize the ABM (Sect. “Tissue remodeling module”).

### Hemodynamics module

**Fig. 3:**
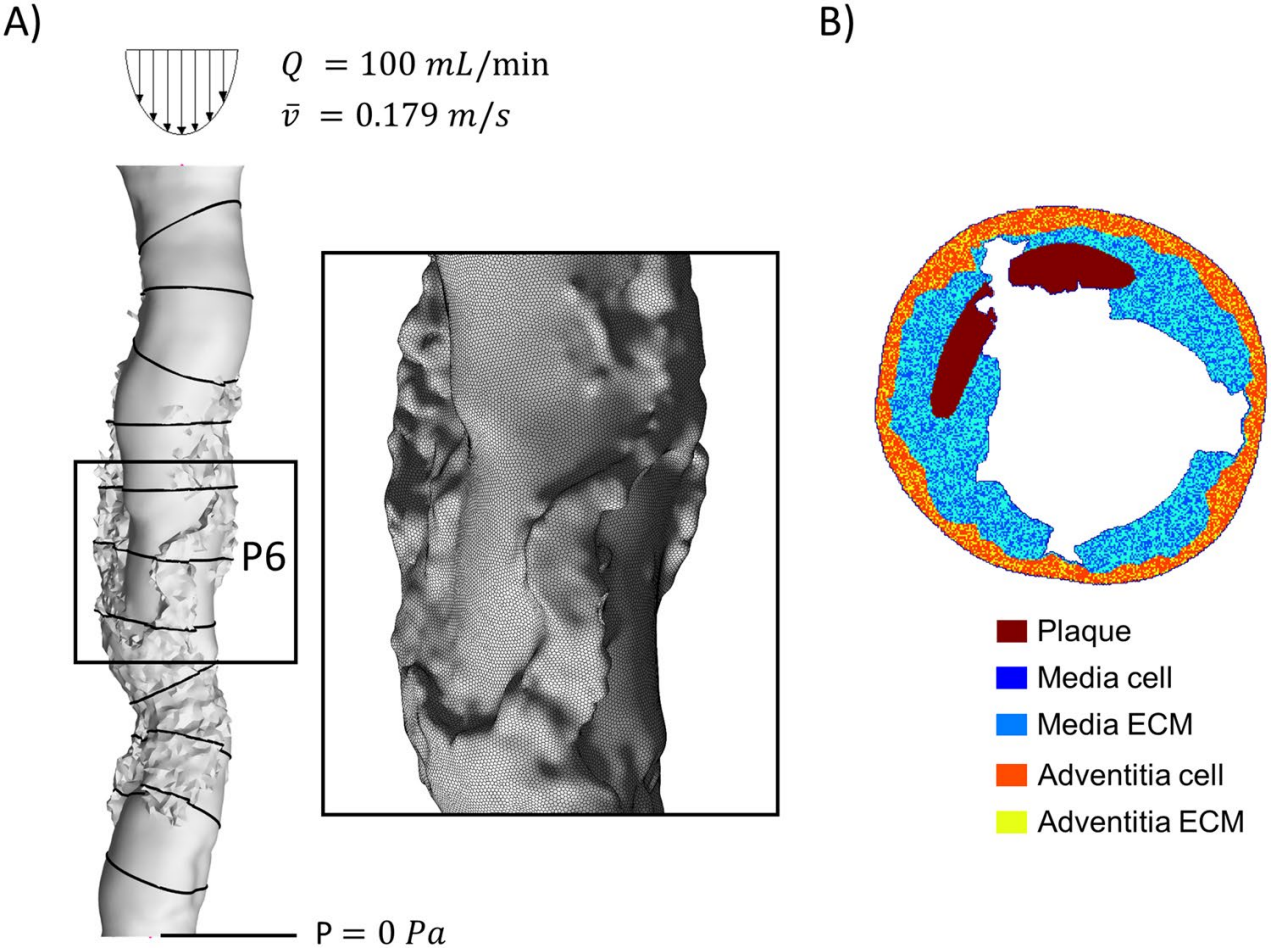
**A** Hemodynamics module. Lumen geometry of the post-PTA configuration of case A used for the computational fluid dynamics (CFD) simulation is shown. The 11 cross-sectional planes selected for the subsequent agent-based model analysis are displayed on the lumen surface. Details of the polyhedral CFD mesh of the wall portion are shown in the black box. **B** Agent-based model of case A, plane 6

The hemodynamics was computed in the post-PTA geometry (first cycle of the framework) and in the remodeled vessel geometry (second cycle of the framework). A polyhedral mesh with five boundary layers of prism elements near the wall was generated in Fluent Meshing (Ansys Inc., Canonsburg, PA, USA) to discretize the fluid domain (Fig. 3A) [28]. Refinement criteria were set based on hardness, proximity and curvature to capture the geometrical features. This resulted in a mesh of ~1,000,000 elements for case A and ~700,000 for case B in the post-PTA models. Steady-state CFD simulations were conducted using Fluent (Ansys Inc.). At the inlet, a parabolic velocity profile was imposed with mean velocity of 0.179 m/s, corresponding to a flow-rate of 100 mL/min, which is typical of SFA [38, 39]. At the outlet, a zero-pressure boundary condition was prescribed. At the walls, assumed as rigid, the no-slip condition was enforced. Blood was modeled as a non-Newtonian Carreau fluid with a density of 1060 kg/m^3^ [40]. Table 4 provides details about the solver settings. At the end of the CFD simulations, the wall shear stress (WSS) was extracted at 2 mm-spaced cross-sectional planes, resulting in 11 planes (Fig. 3A).

**Table 4:** List of the computational fluid dynamics solver settings

Type	Ansys Fluent—pressure-based
Pressure-velocity coupling method	Coupled
Spatial discretization scheme–gradient	Least squares cell based
Spatial discretization scheme–pressure	Second order
Spatial discretization scheme–momentum	Second order upwind
Convergence criterion for the global residuals	5 × 10^−5^

### Tissue remodeling module

The tissue remodeling module consisted of the 2D ABM simulation of post-PTA arterial wall remodeling and the subsequent reconstruction of the 3D remodeled vessel from the ABM outputs. The 2D ABM, adapted from [28], was implemented in Matlab for each of the 11 vessel cross-sections and simulated the in-plane arterial wall remodeling at cell-tissue scale in response to the PTA-induced wall damage and WSS, by replicating cellular dynamics (Fig. 3B).

Each ABM plane was generated on a 300 × 300 hexagonal grid through the application of a 3D to 2D transformation to the nodal coordinates extracted from the post-PTA geometry. Besides coordinate translation and rotation, a scaling of 0.0375 mm/ABM site was set, thus assuming 1.5 cells per ABM site (SMC diameter of ~25 µm [41]). The ABM comprised the media and adventitia layers, as well as the plaque, according to the node-layer association of the 3D geometry. Also within the ABM, the media layer was assumed to embed the intima layer [19]. SMCs, collagen, and elastin composed the media layer (with SMC/ECM ratio of 0.72 [42] and collage/elastin ratio of 0.63 [43]). Fibroblast and collagen composed the adventitia (with fibroblast/collagen ratio of 0.43 [44]).

The ABM was initialized with damage- and WSS-based inputs (*Dinput* and *WSSinput*, respectively), driving the probabilistic behavioral rules that govern cellular activities, as detailed in Table 5 and explained below. *Dinput* represented the inflammatory trigger resulting from the injury induced by PTA. It was assumed that a higher PTA-induced damage would result in a more pronounced local inflammatory response, consequently leading to an intensification of cellular activities. Specifically, the damage computed through the PTA module was assigned to each agent and used to locally weigh a generic post-intervention time-varying inflammatory curve [45] (Supplementary Figure S2). This curve exhibited a peak around day 3 and vanished at 1 month after the intervention. *WSSinput* incorporated the modulating effect of WSS on SMC dynamics, according to which increased SMC synthetic and proliferative activities are found at low WSS regions [8]. Specifically, (i) the WSS computed through the hemodynamics module was assigned to each lumen wall agent, (ii) a sigmoid-shaped function was formulated to represent the inverse relationship between WSS and the hemodynamic triggering input (i.e., low WSS corresponded to a high input), and (iii) the input was propagated across the thickness of the media layer using a sinusoidal function (Table 5). Consequently, at each time step, cellular activities in the media were governed by *Dinput* (up to 1-month follow-up, given the duration of inflammation) and *WSSinput*. Further details on *Dinput* and *WSSinput* definition, as well as their governing parameters, are reported in the Supplementary Materials.

**Table 5:** Agent-based model input definition and probability equations

*Dinput* definition	${Dinput}^{h}= {damage}^{h}\times Inflammation(t-delay)$
*WSSinput* definition	${D(WSS)}^{i}= -\frac{1}{1+{e}^{{L}_{1}\left({WSS}^{i}-{L}_{2}\right)}}+1$
	$${WSSinput}^{h}= \left\{\begin{array}{c}{D(WSS)}^{i} \,\, lumen\\ \sum_{i}D{(WSS)}^{i}\times Amp\times (1+{\text{cos}}\left(\pi \frac{x}{dist}\right)) \,\,intima\end{array}\right.$$
Cellular activity probabilistic rules	$$media: \left\{\begin{array}{c}{p}_{migration}^{h}=\frac{1}{{distance}_{min}(h, {site}_{mig}))}\\ {p}_{division}^{h}= {\alpha }_{1}+{\alpha }_{2}{WSSinput}^{h}{+ \alpha }_{3}{Dinput}^{h} \\ {p}_{apoptosis}^{h}= {\alpha }_{1} \\ {p}_{ECMproduction}^{h}= {\alpha }_{4}+{\alpha }_{5}{WSSinput}^{h}{+ \alpha }_{6}{Dinput}^{h}\\ {p}_{ECMdegradation}^{h}= {\alpha }_{4}/{\beta }_{med}\\ \end{array}\right.$$
	$$adventitia: \left\{\begin{array}{c}{p}_{division}^{h}= {p}_{apoptosis}^{h}= {\alpha }_{1}\\ {p}_{ECMproduction}^{h}= {\beta }_{adv}\cdot {p}_{ECMdegradation}^{h}= {\alpha }_{4}\end{array}\right.$$

*h:* agent site; *Dinput*: damage-based input; *damage*: value of damage computed from PTA simulations; *Inflammation*: inflammatory curve; *t*: time; *delay*: 3 days; *i*: agent site on the lumen wall; *D(WSS)*: level of endothelial dysfunction; *WSS*: wall shear stress; *L*_*1*_: slope of the logistic curve; *L*_*2*_: WSS value at which D(WSS) = 0.5; *WSSinput*: WSS-based input; *Amp*: scaling factor; *x*: distance of the intima site *i* from the lumen wall, *x < dist*; *dist* = distance parameter; ${p}_{migration}^{h}$: probability of cell migration; ${distance}_{min}$: minimum distance; ${site}_{mig}:$ potential migration site; ${p}_{division}^{h}$: probability of cell mitosis; ${p}_{apoptosis}^{h}$: probability of cell apoptosis; ${p}_{ECMproduction}^{h}$: probability of extracellular matrix (ECM) production; ${p}_{ECMdegradation}^{h}$: probability of ECM degradation; ${\alpha }_{1}$, ${\alpha }_{2}$, ${\alpha }_{3}$, ${\alpha }_{4}$, ${\alpha }_{5}$, ${\alpha }_{6}, {\beta }_{med}$, ${\beta }_{adv}$: parameters driving agent probabilities

Probabilistic behavioral rules were defined to simulate cell migration, mitosis/apoptosis, and ECM production/degradation (Table 5, with values of the parameters listed in Table 6). Cell migration was implemented to simulate the recruitment of cells at arterial wall regions that underwent lacerations during the expansion of the angioplasty balloon. Potential migrating cells were those SMCs belonging to the innermost layer of the media (i.e., lining the lumen) and presenting with high damage values after the balloon expansion. The sites for potential migration were the lumen sites lining the lacerated plaque. The probability of SMC migration ($${p}_{migration}^{h})$$ was defined to be inversely proportional to the minimum distance between the potential migrating SMC and the nearest migration site (Table 5). Migration was halted when one of the following conditions was satisfied: either all potential migrating SMCs have completed their migration, or all available potential migrating sites have been occupied. Cell mitosis/apoptosis $$({p}_{division}^{h}$$ and $${p}_{apoptosis}^{h}$$, in Table 5) and ECM production/degradation ($${p}_{ECMproduction}^{h}$$ and $${p}_{ECMdegradation}^{h}$$, in Table 5) were set such that cell mitosis and ECM production in the media depended on *Dinput* and *WSSinput* (i.e., the higher the triggering inputs, the higher the probabilities), while baseline activities were maintained in the adventitia layer. The ABM parameters were set according to previous works [17–19, 28, 46–48] and tuned according to the following criteria: (i) maintenance of the homeostatic condition in the absence of stimuli and (ii) maintenance of the physiological ECM/SMC ratio (i.e., the ratio between the final and initial ECM/SMC ratio fell within the range [0.5 1.5], as in [17, 28]), in agreement with the hypothesis that the relative arterial tissue composition does not undergo extreme changes over time within the first post-operative months [49–51]. Specifically, while $${\alpha }_{1}, {\alpha }_{4}$$ and β_adv_, driving the baseline dynamics, were set as in [17–19, 28, 46–48], β_med_ was calibrated to guarantee an equilibrium of the ECM dynamics in the media layer, under homeostatic condition. Moreover, to compensate the greater ECM production over cell proliferation in the media layer, which is due to the higher baseline ECM content with respect to the cellular one, the values of the parameters related to the cellular dynamics ($${\alpha }_{2}$$ and $${\alpha }_{3}$$) were set to be higher than those related to the ECM dynamics ($${\alpha }_{5}$$ and $${\alpha }_{6}$$).

**Table 6:** Agent-based model parameters

Parameter	*L*_*1*_	*L*_*2*_	Amp	Dist	${\alpha }_{1}$	${\alpha }_{2}$	${\alpha }_{3}$	${\alpha }_{4}$	${\alpha }_{5}$	${\alpha }_{6}$	*βmed*	*βadv*
Value	-7.55	1	0.14	15	0.05	0.11	0.66	0.008	0.0413	0.21	1.85	2.5

The ABM scheme previously developed in [17–19, 28, 46, 47] was applied in this study. Briefly, agent dynamics were desynchronized by initializing each agent with a random time within the agent biological cycle T_agent_ (T_cell_ = 24 hours and T_ECM_ = 4 hours), and updating it at each 2-hour time step. At each time step, agents that were potentially active (i.e., those reaching the end of their biological cycle) were identified and examined to determine whether a biological event (i.e., cell mitosis/apoptosis or ECM production/degradation) took place. The event occurred only if the agent-specific event probability exceeded a randomly generated number. Cell mitosis and ECM production determined agent generation, while cell apoptosis and ECM degradation determined agent removal. Agent generation/removal within the arterial wall was oriented inward in the media (i.e., implying media area change, with subsequent lumen area change) and oriented outward in the adventitia (i.e., implying adventitia area change, without lumen area change). Finally, to maintain regular contours, smoothing algorithms were applied [28].

To account for the ABM stochasticity, three simulations were run for each plane up to the coupling period (i.e., 1 month), when the ABM simulations were stopped to reconstruct the 3D lumen geometry. To this aim, for each plane the output configuration among the three that minimized the root-mean-square deviation of the lumen contour from the average one was considered and the follow-up arterial lumen surface was reconstructed by lofting the lumen contour of the selected ABM outputs [46].

### Statistical analysis

Mann–Whitney *U*-test was adopted to compare the lumen area distributions (e.g., the lumen area of case A and B at different time points) and statistical significance was assumed for *p*-values < 0.05. The statistical analysis was conducted in Matlab.

## Results

**Fig. 4:**
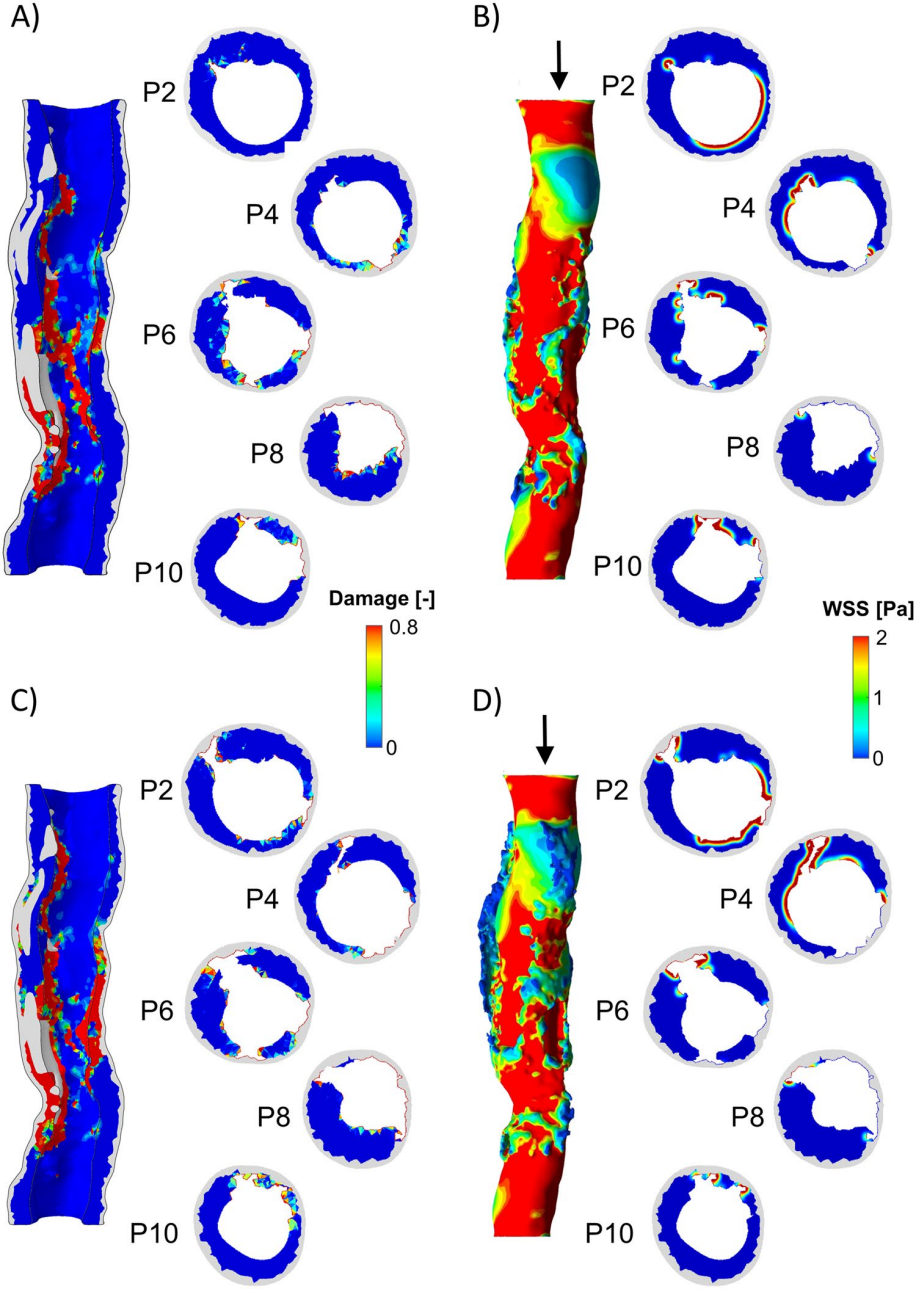
Results of the percutaneous transluminal angioplasty (PTA) and hemodynamics modules. **A** Damage map in the 3D model and for 5 explanatory planes obtained from the PTA module, for case A. **B** Wall shear stress (WSS) contour in the 3D model and for 5 explanatory planes obtained from the hemodynamics module at time 0 (after the PTA), for case A. **C** Damage map in the 3D model and for 5 explanatory planes obtained from the PTA module, for case B. **D** WSS contour in the 3D model and for 5 explanatory planes obtained from the hemodynamics module at time 0 (after the PTA), for case B

**Fig. 5:**
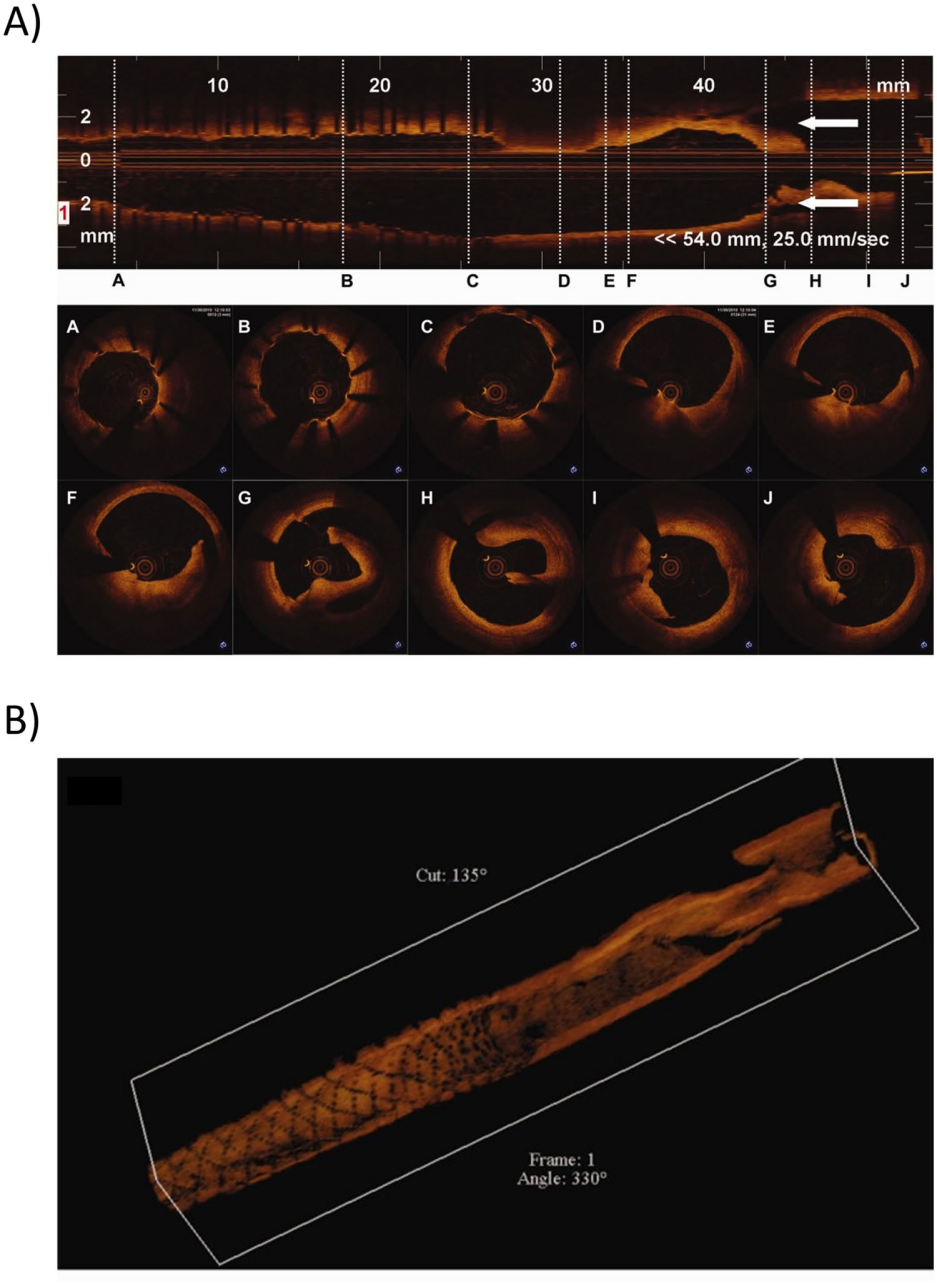
**A** Optical coherence tomography of superficial femoral artery, showing arterial wall dissection following percutaneous transluminal angioplasty. At the top, the longitudinal view is reported, with the corresponding cross-sectional views (dashed white lines A–J) at the bottom. Dissection flaps are visible from panel D to J. **B** Three-dimensional reconstruction of frequency domain optical coherence tomography images. *Partially adapted with permission from Catheterization and Cardiovascular Interventions 2013, 81(3):568-72. Stefano GT et al. Imaging a spiral dissection of the superficial femoral artery in high resolution with optical coherence tomography-seeing is believing. *https://doi.org/10.1002/ccd.24292*. * © 2012 Wiley Periodicals, Inc.

The virtual PTA procedure performed in the diseased SFA model resulted in a median increase in lumen area of 26% in case A and 43% in case B. More in detail, commencing from a pre-operative area of 6.19 [5.28–7.40] mm^2^ (with minimum lumen area of 2.45 mm^2^), the PTA procedure led to a post-PTA area of 7.82 [6.48–8.61] mm^2^ (with minimum lumen area of 5.26 mm^2^) in case A and 8.84 [6.98–9.93] mm^2^ (with minimum lumen area of 5.32 mm^2^) in case B. Moreover, considering a target lumen area of 8.55 mm^2^ (associated with the inlet diameter of 3.3 mm), the percentage of residual stenosis on the lumen area following the PTA procedure was 38.5% and 37.8% for case A and B, respectively. The expansion of the balloon induced damage in the arterial wall, with high values (> 0.8) observed in various regions along the length of the artery, as shown in Fig. 4 (panels A and C). In the regions with high damage, lacerations and dissections occurred as result of the element deletion strategy implemented in Abaqus. Higher levels of damage were observed in case B in comparison to case A, due to the greater expansion of the balloon in case B (Fig. 4). Consequently, larger dissections were evident in case B when compared to case A, resulting in a larger post-operative median lumen area (*p* < 0.05). The presence of dissected and lacerated arterial wall segments aligned with prior OCT and experimental findings in SFAs [52, 53], as reported in Fig. 5. In particular, the laceration and flap obtained in P8 for case A (Fig. 4A) and P4 for case B (Fig. 4C) highly resembles the OCT cross-sections F, G, and H (Fig. 5A) while P2, P4, P6, and P10 of case A (Fig. 4A) and P2, P6, P8, and P10 of case B (Fig. 4C) show high similarities with the OCT cross-sections E, I and J (Fig. 5A). Moreover, similarly to the OCT findings (Fig. 5B), the obtained laceration developed longitudinally (Fig. 4B–D).

The presence of dissections and lacerations in the post-intervention arterial configuration subsequently affected the hemodynamics and the WSS (Fig. 4B–D). Indeed, in both cases A and B, low WSS (WSS < 1 Pa) were found in correspondence of the lacerations. Moreover, as the lacerations generates geometrical irregularities, the larger the lacerations, the lower the WSS. Thus, overall, case B exhibited lower WSS values than case A.

**Fig. 6:**
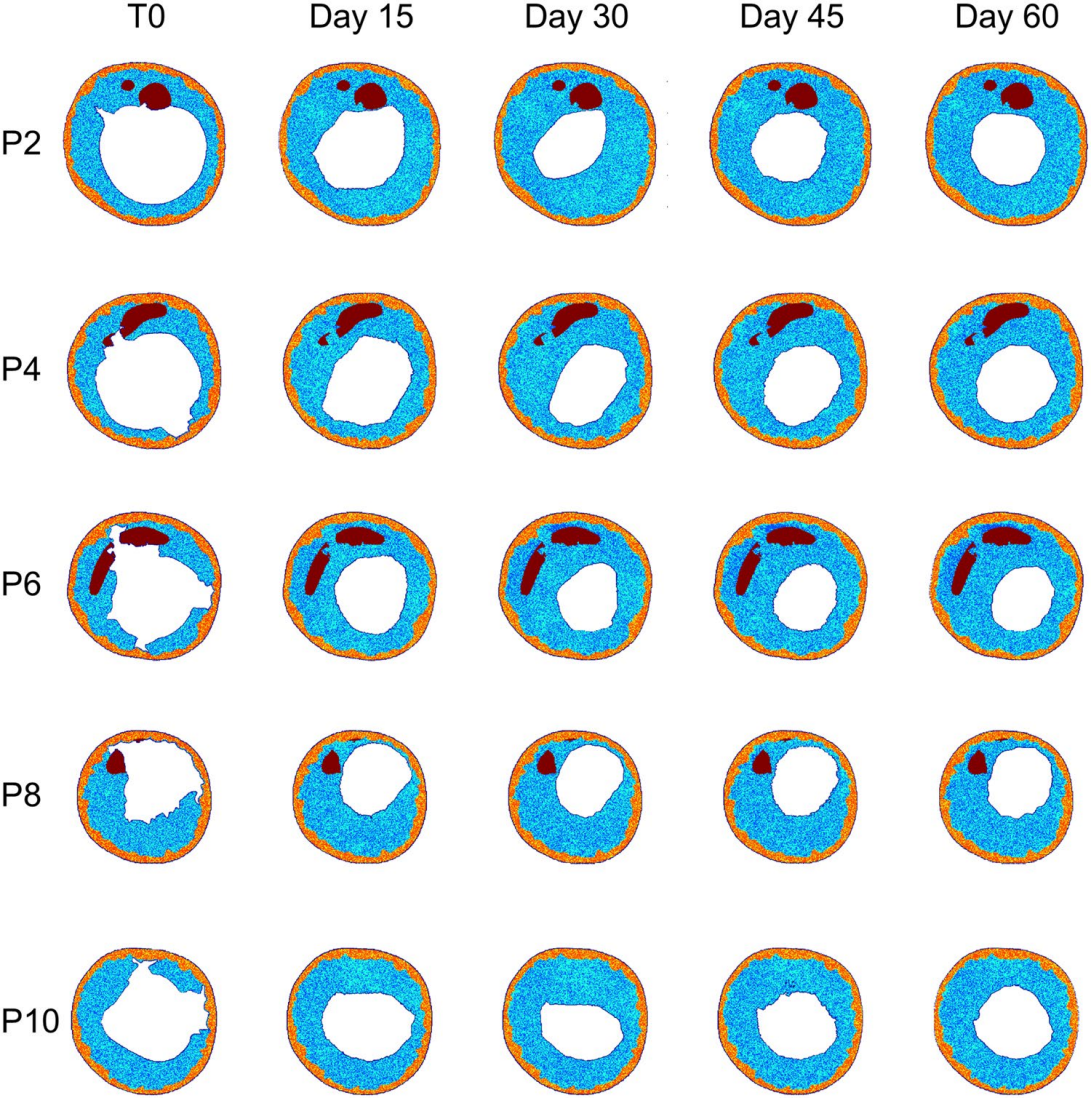
Results of the tissue remodeling module for case A. Temporal evolution of the agent-based models (ABM) of 5 explanatory planes (planes 2, 4, 6, 8, and 10) along 2 simulated months. For each ABM plane, the monthly output was retrieved from 1 out of 3 ABM simulations, namely the one presenting the lumen configuration minimizing the root-mean-square deviation

**Fig. 7:**
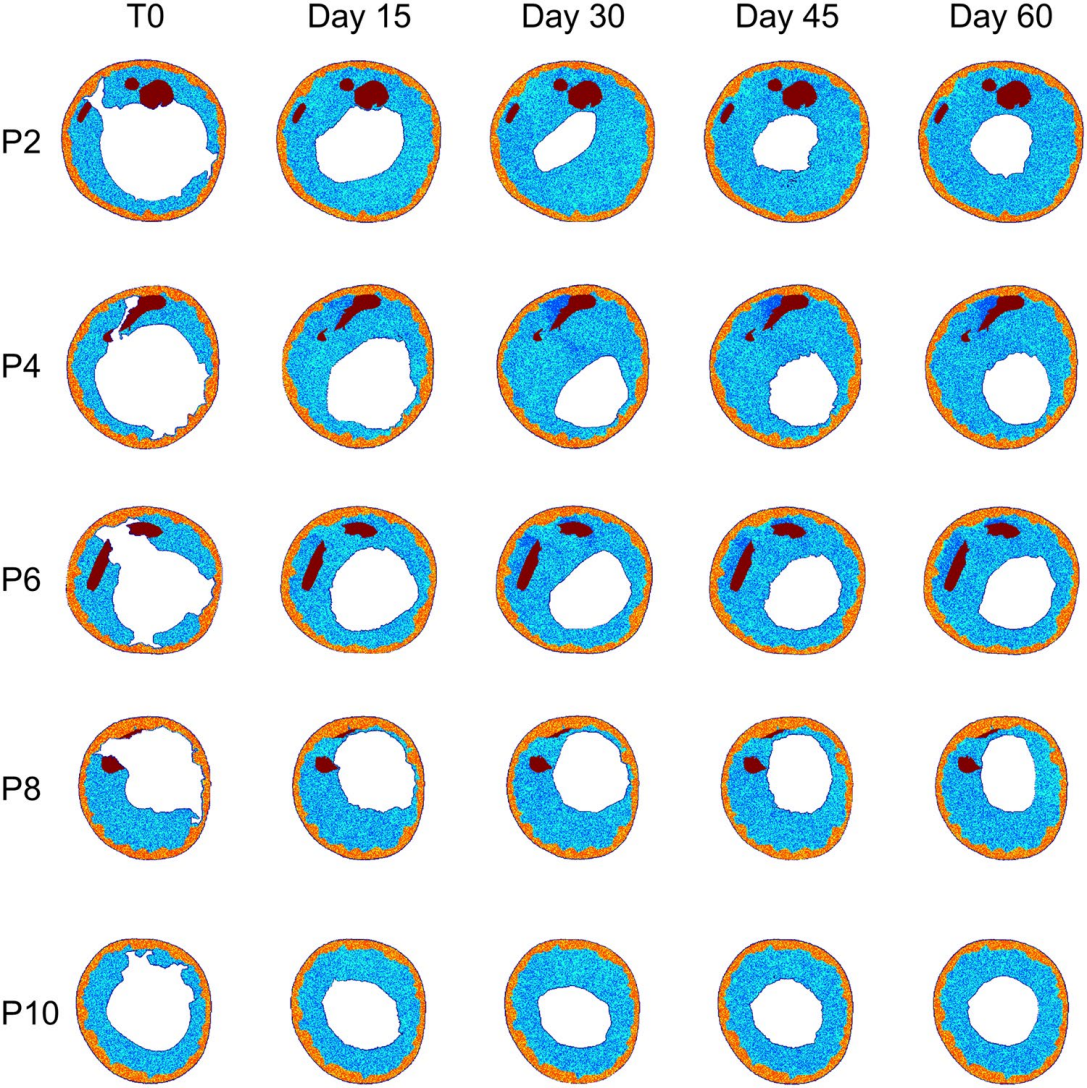
Results of the tissue remodeling module for case B. Temporal evolution of the agent-based models (ABM) of 5 explanatory planes (planes 2, 4, 6, 8, and 10) along 2 simulated months. For each ABM plane, the monthly output was retrieved from 1 out of 3 ABM simulations, namely the one presenting the lumen configuration minimizing the root-mean-square deviation

**Fig. 8:**
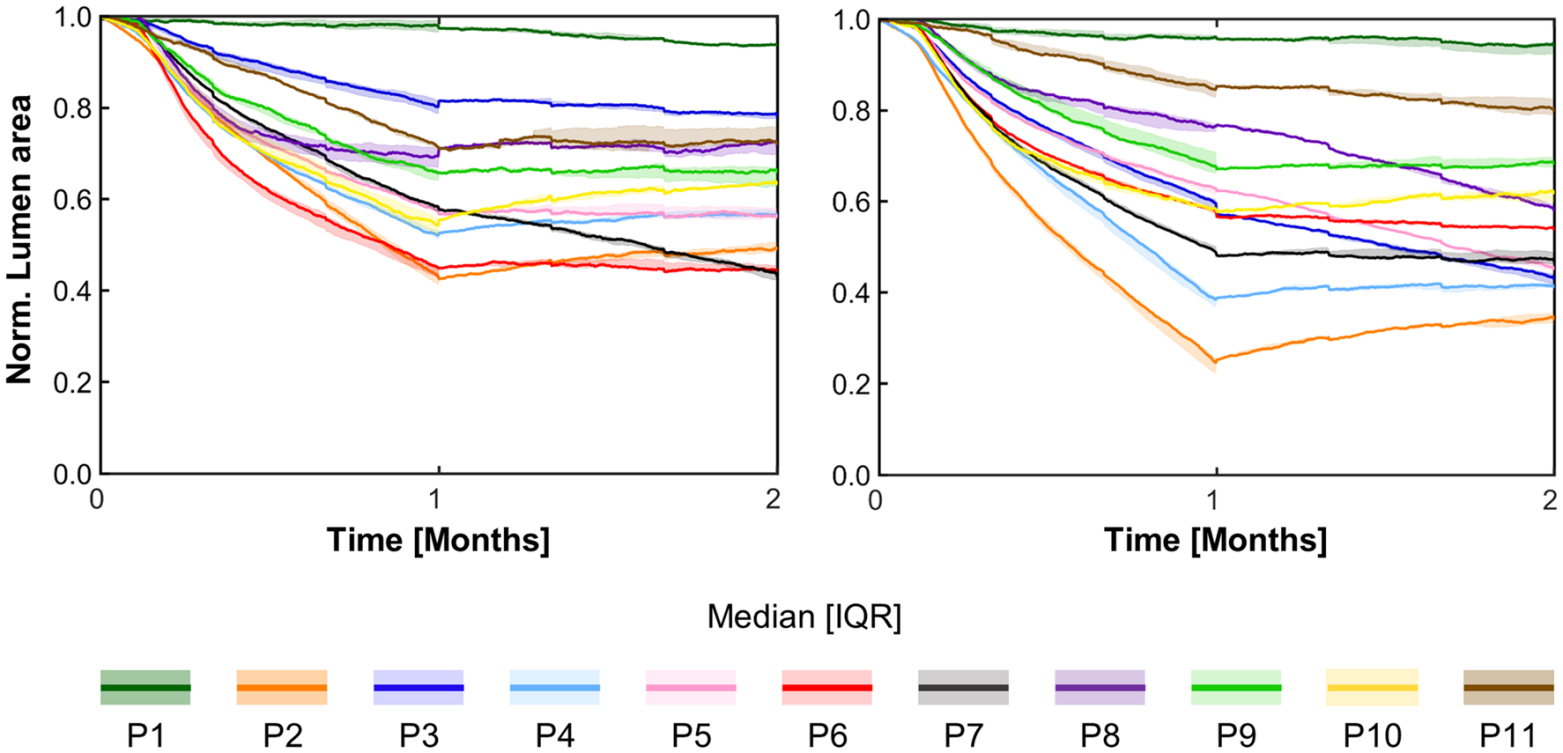
Normalized lumen area over time (median and interquartile range) for the 11 agent-based model planes (P1–P11) of case A (left) and B (right)

Damage and WSS served as drivers for post-operative arterial wall remodeling along 2 post-operative months, with WSS being updated in the remodeled vessel geometry after 1 simulated month. Figure 4 illustrates *Dinput* and *WSSinput* for 5 explanatory ABM planes (i.e., plane 2, 4, 6, 8, and 10) for both cases A and B. The ABM evolution of these planes over the period of 2 post-operative months is shown in Fig. 6 (case A) and Fig. 7 (case B), while Fig. 8 details the normalized lumen area trend of all the 11 planes. As depicted in Figs. 5 and 6, the ABM cross-sections at time 0 were characterized by lacerations in both the media layer and plaque region. However, by day 15, these regions had mostly undergone recovery. This recovery was attributed to the fact that these regions were characterized by high *Dinput* and *WSSinput*, which facilitated cell migration, proliferation, and ECM production in the media. The growth process persisted in all the planes until day 30, at which point a shift in driving inputs occurred. Specifically, (i) *Dinput* decreased to 0 because of the inflammatory response, and (ii) *WSSinput* was updated within the remodeled geometry. Consequently, only a few regions remained exposed to a non-zero *WSSinput*. As a result, most planes experienced either a stabilization in growth or a reduction in the growth rate. This observation highlights the effect of the coupling between the tissue remodeling and hemodynamics modules, consisting in capturing the mutual interaction between arterial lumen remodeling and fluid dynamics. As shown in Fig. 8, for case A, plane 2 and plane 6 experienced the greatest restenosis at day 30, resulting in a lumen area reduction of 57% and 55% respectively, with stabilization occurring during month 2. Conversely plane 7, which presented a lumen area reduction of 42% at day 30, achieved a lumen area reduction of 57% at day 60. As regards case B, overall, a more severe restenosis was observed compared to case A. Notably, plane 2 showed the greatest lumen area reduction, reaching 75% at day 30 and 66% at day 60. The distinctive behavior of this plane, which underwent a slight recovery during the second month, has been previously observed in the patient-specific framework of in-stent restenosis developed by our group [18]. This behavior is attributed to the assumed constraint on the ratio between ECM and cells, according to which the initial ECM/SMC ratio should be preserved throughout the restenosis process, with admissible oscillations of the ECM/SMC ratio normalized to the initial value in the range [0.5 1.5]. Accordingly, for each plane, the ECM and SMC growth rate was similar throughout the entire remodeling process, thus, preserving the ECM/SMC ratio (Supplementary Fig. S4). Differently, for plane 2 of case B, the ECM increased with a growth rate greater than that of SMC in the first month, leading to an increase of the normalized ECM/SMC ratio, which was then restored through the slight decrease of ECM in the second month (Supplementary Fig. S4, panel B), responsible for the lumen area increase.

**Fig. 9:**
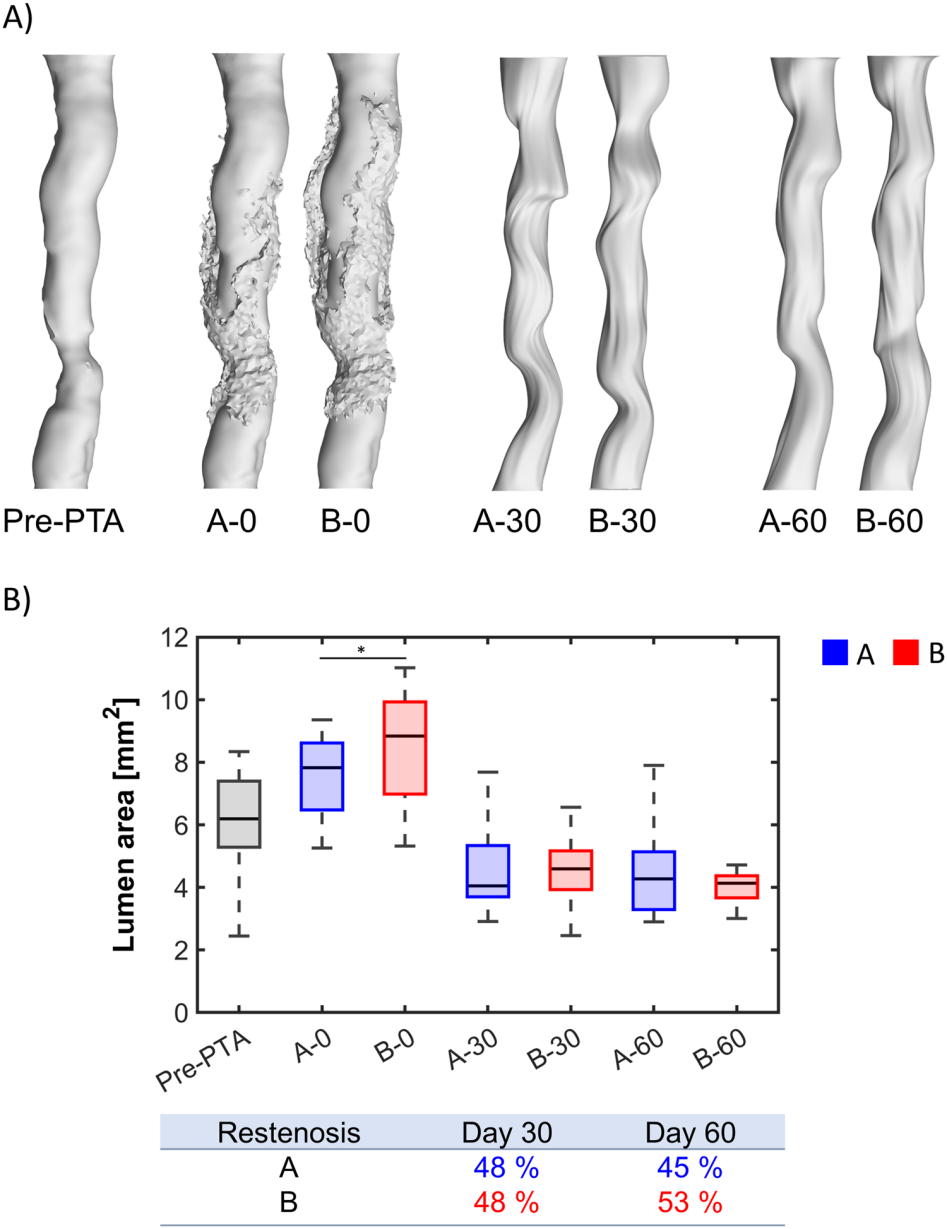
**A** Comparison between the lumen geometries before the intervention (pre-PTA), immediately after the intervention (time 0) and at day 30 and 60 after the intervention, for cases A and B. **B** Boxplots of the lumen area before the intervention (pre-PTA), immediately after the intervention (time 0) and at day 30 and 60 after the intervention, for cases A and B. Statistical difference is shown only for the comparison between case A and case B *p < 0.05

Figure 9 shows the 3D lumen geometries (panel A) and the boxplots (panel B) of the lumen area before the intervention (pre-PTA), immediately after the intervention (post-PTA, day 0) and at day 30 and day 60, for cases A and B. Although case B presented a significantly larger lumen area at day 0 compared to that of case A (*p* < 0.05), no significant differences were found between the lumen areas of the two cases at day 30 and day 60. At day 30, both cases A and B underwent a median restenosis of 48%, with a minimum lumen area of 2.91 mm^2^ and 2.46 mm^2^, respectively. At day 60, case A exhibited a median restenosis of 45%, with minimum lumen area of 2.90 mm^2^, whereas case B underwent a median restenosis of 53%, with a minimum lumen area of 3.00 mm^2^. Tables 7 and 8 provide a summary of the global and minimum lumen areas at the follow-up times for both cases A and B.

**Table 7:** Lumen area (median [interquartile range]) of cases A and B

Case	Pre-PTA [mm^2^]	Post-PTA [mm^2^]	Day 30 [mm^2^]	Day 60 [mm^2^]
A	6.19 [5.28–7.40]	7.82 [6.48–8.61]	4.05 [3.70–5.34]	4.27 [3.29–5.14]
B		8.84 [6.98–9.93]	4.59 [3.93–5.17]	4.13 [3.67–4.37]

**Table 8:** Minimum lumen area of cases A and B

Case	Pre-PTA [mm^2^]	Post-PTA [mm^2^]	Day 30 [mm^2^]	Day 60 [mm^2^]
A	2.45	5.26	2.91	2.90
B		5.32	2.46	3.01

## Discussion

This study has introduced a patient-specific, multiscale agent-based modeling framework for investigating restenosis following PTA in SFAs. The framework replicates the post-intervention arterial wall remodeling in response to the PTA-induced wall injury and altered hemodynamics, through the combination of three modules, namely (i) the PTA module (finite element simulation), (ii) the hemodynamics module (CFD simulations), and (iii) the tissue remodeling module (ABM simulations). The overall structure of the framework builds upon a pilot study conducted by the authors [28], where a proof-of-concept application was demonstrated on an idealized diseased SFA model. Herein, the significant advancements introduced in the PTA module along with the refinement of the ABM and the application of the framework to a patient-specific SFA model, mark substantial progress in the in silico modeling of restenosis, further confirming the considerable potential of the proposed framework, as elaborated below.

While the overall underlying hypotheses were built on our previous works [17, 18, 28], the primary innovation lies in the integration of a sophisticated finite element model of the PTA procedure within the framework. This model incorporates anisotropic hyperelastic arterial tissues embedding a damage model, utilizing material properties derived from human SFA data. Moreover, the inclusion of the element deletion approach in the PTA simulation is a noteworthy addition. This, in conjunction with the damage model, enables the replication of PTA-induced lacerations and dissections, phenomena observed in real case scenarios [52]. It is important to note that due to the unavailability of post-intervention intravascular imaging data, a strict validation of these results was not feasible. However, the high resemblance between the post-intervention vessel configurations obtained in this study (Fig. 4) and the data reported by Stefano et al. [52] and in Fig. 5, which were derived from post-intervention examinations employing OCT, emphasizes the validity of the simulation in providing realistic post-intervention scenarios.

As extensively discussed elsewhere [11], several multiscale agent-based modeling frameworks of restenosis following endovascular procedures (mainly implying stent deployment) have been developed to date. However, these prior frameworks were (i) mainly focused on either the effects of the intervention-induced damage [16, 24–27] or the hemodynamic alteration [14, 15, 20–23] on the arterial wall response and (ii) applied to idealized case scenarios. To the best of the authors’ knowledge, the sole application of a multiscale agent-based modeling of vascular adaptation to patient-specific cases is the in-stent restenosis model recently developed by Corti et al., [17, 18]. Nevertheless, in that application, only the arterial lumen geometry was derived from patient-specific images, with the outer wall assumed to be circular, and uniform tissue composition without plaque components considered. Herein, although a two-layer arterial wall was modeled (due to the impossibility of identifying the internal elastic lamina from OCT images), a more accurate vessel reconstruction was pursued. This involved incorporating image-derived geometries of the luminal wall, external elastic lamina and outer wall, as well as accounting for plaque components.

Overall, the current study substantially enhances our previously proposed multiscale agent-based modeling framework of restenosis [28], enabling a more realistic representation of the complex, multiscale and multifactorial network of the underlying mechanobiological processes occurring after PTA in a patient-specific SFA. Specifically, the framework, enhanced by the application to a patient-specific setting and a sophisticated modeling of the arterial wall mechanical properties, was able to capture major mechanobiological events characterizing the vascular adaptation process after PTA, including: (i) the presence of lacerated and dissected arterial tissue resulting from balloon expansion, (ii) the early cellular activation as a response to the PTA-induced injury, characterized by prompt cellular migration to the injured regions and healing through cell synthetic and proliferative activities, (iii) the exacerbated intimal growth (cell proliferation and ECM production) leading to subsequent lumen area reduction, as a consequence of the maladaptive healing response, triggered by the sustained damage-related inflammatory condition and hemodynamic alteration persisting up to 1 post-operative month, and (iv) a stabilization of the lumen area occurring after 1 month, attributed to the recovery of the inflammatory state and the changes in the hemodynamic environment.

The investigation of the impact of two different balloon expansion conditions, namely maximum expansion diameter of 5.2 mm (case A) and 6.2 mm (case B), on the arterial wall response provided insights into potential applications of the proposed framework in the context of “in silico medicine”. Interestingly, despite the significantly larger post-PTA lumen area resulting from the greater balloon expansion in case B, which might initially appear as a more favorable condition, the findings indicated that there were no significant differences in lumen areas between cases A and B at both 1 and 2 months after the intervention. While these results did not undergo validation, this application demonstrated that, after a proper calibration and validation, the framework could be applied to provide valuable insights into the outcome of the intervention, enabling for example the optimization of the procedural steps within a patient-specific context and tailoring the procedure to obtain more favorable outcomes. Moreover, the framework could be used to enhance our understanding of the pathophysiological mechanisms underlying restenosis following PTA. Specifically, by applying the same intervention conditions to various patient-specific cases, it becomes possible to identify individual pathological pathways, highlighting patient-specific factors associated with the restenosis process. To these aims, incorporating patient-specific scenarios enriched with precise modeling of arterial wall composition and mechanical properties is essential. Accordingly, the present work paves the way towards the definition of a “digital twin” of the PTA procedure and post-intervention arterial response, providing a substantial contribution in the context of in silico personalized medicine. Finally, as already demonstrated in our previous investigations on in-stent restenosis in patient-specific SFAs [17, 18], the inherent modularity of the general framework allows for the integration of multi-omics data, potentially resulting in further advancements within the field of cardiovascular personalized medicine.

A major limitation of the study relies in the lack of calibration and validation. Due to the unavailability of follow-up data, it was not possible to assess the validity of the specific obtained results. However, the PTA-induced arterial wall lacerations and dissections obtained as output of the finite element simulation of PTA (Fig. 4) were consistent with intravascular observations in human SFAs [52] (Fig. 5). Furthermore, an earlier clinical investigation identified a 1-month restenosis rate of up to 12.7% in coronary arteries treated with PTA [54] (with restenosis defined as a reduction of > 50% in lumen area). This suggested that substantial changes in lumen area, similar to those simulated in the current framework (~48%), may occur in vivo. Additionally, the simulated two-phase lumen area trend resembled the one observed in SFAs treated with angioplasty and self-expanding stent implantation [55]. This trend was also consistent with the current understanding of the pathobiological mechanisms of restenosis, characterized by high lumen area reduction due to injury-activated synthetic SMC proliferation within the first post-operative month, after which re-endothelialization usually occurs [56]. In light of the aforementioned considerations, while quantitative validation remains unattainable, the validity of the model assumptions and, consequently, of the overall qualitative results can be reasonably affirmed. In the future, to broaden the framework's applicability for refining patient-specific treatments or assessing diverse patient responses, it is essential to calibrate and validate the framework. For this purpose, the pipeline proposed in [17, 18] can be employed, provided that longitudinal patient data are available. This methodology involves (i) conducting a sensitivity analysis on the ABM parameters and on the ABM-CFD coupling frequency to identify the driving parameters, (ii) calibrating the identified parameters through a combined surrogate modeling—genetic algorithm optimization approach, and (iii) validating the framework by applying it to a different patient case. Other future developments of the proposed framework include the application to stented femoral arteries, or the implementation of the thrombosis mechanisms within the ABM, as this process represents a major drawback of PTA in lower limb arteries [57].

In conclusion, the proposed multiscale agent-based modeling framework, by integrating a sophisticated finite element simulation of the PTA procedure, CFD simulations, and an ABM of cellular dynamics, provided insights into (i) the post-PTA artery condition, characterized by extremely injured tissue, and (ii) the post-intervention remodeling process, characterized by an initial recovery of the injured regions, followed by an intense cellular activity leading to high lumen area reduction ad 1 month, and a final stabilization at 2 months. The similarity of the obtained results with clinical observations in treated SFAs suggested the potentiality of the developed framework in capturing patient-specific mechanobiological events occurring after PTA intervention.

### Supplementary Information

Below is the link to the electronic supplementary material.

Supplementary file1 (PDF 732 KB)
