# Supplementary material for: Impact of Tissue Damage and Hemodynamics on Restenosis Following Percutaneous Transluminal Angioplasty: A Patient-Specific Multiscale Model
Source: Ann Biomed Eng. 2024 May 3;52(8):2203–20. doi: 10.1007/s10439-024-03520-1 (PMC11247064; doi:10.1007/s10439-024-03520-1)
Supplement: Supplementary file 1 — Supplementary file1 (PDF 732 KB) [file 10439_2024_3520_MOESM1_ESM.pdf]

Impact of tissue damage and hemodynamics on restenosis following percutaneous transluminal angioplasty: a patient-specific multiscale model

Anna Corti[1], Matilde Marradi[2,3], Cemre Çelikbudak Orhon[4], Francesca Boccafoschi[5], Philippe Büchler[6], Jose F. Rodriguez Matas[2], Claudio Chiastra[7]

1. Department of Electronics, Information and Bioengineering, Politecnico di Milano, Milan, Italy
2. Laboratory of Biological Structure Mechanics (LaBS), Department of Chemistry, Materials and Chemical Engineering “Giulio Natta”, Politecnico di Milano, Milan, Italy
3. MERLN Institute for Technology-Inspired Regenerative Medicine, Department of Cell Biology-Inspired Tissue Engineering, Maastricht University, Maastricht, The Netherlands
4. Laboratory of Hemodynamics and Cardiovascular Technology, Institute of Bioengineering, Ecole Polytechnique Fédérale de Lausanne, Lausanne, Switzerland
5. Department of Health Sciences, University of Piemonte Orientale "A. Avogadro", Novara, Italy
6. ARTORG Center for Biomedical Engineering Research, University of Bern, Bern, Switzerland
7. Polito[BIO]Med Lab, Department of Mechanical and Aerospace Engineering, Politecnico di Torino, Turin, Italy

***Address for correspondence:**

Anna Corti, PhD

Department of Electronics, Information and Bioengineering, Politecnico di Milano, Via Golgi 39, 20131 Milan, MI, Italy

anna.corti@polimi.it – ORCID ID: 0000-0001-9603-7825

SUPPLEMENTARY METHODS

Fitting on experimental data

A sample of distal superficial femoral artery (SFA) tissue was provided by the Vascular Surgery Unit, Hospital Maggiore, Novara (Italy). All data and samples were collected from 10 donors subjected to open surgical repair for popliteal artery prosthetic substitution and correctly informed for the use of excessive material for diagnostic and research purpose according to the local institute's regulation and policies based on Declaration of Helsinki (AVATAR, 1.0 – protocol 208/CE – CE 43/18, date of approval: 6 April 2018). Supplementary Table S1 shows the clinical features for the patients recruited [1].

Uniaxial tensile tests were performed using a bioreactor with force measurement TC3F. From the SFA sample, four specimens were obtained, two in the longitudinal direction and other two in the circumferential direction. After preconditioning, the specimens were stretched with strain rate of 0.1%/s until failure. Reaction forces were recorded. Strain values were calculated as displacement values divided by the initial length of the sample. Then, stretch values were found with addition of 1 to the strain values so that stretch value of 1 represents initial condition without any load. First Piola-Kirchhoff stress $\boldsymbol{P}$ was considered and computed as current load divided by initial cross-section area of the sample. Figure S1 shows the average circumferential and longitudinal stress-stretch response of human distal SFA specimens after eliminating the preconditioning and post-failure effects.

First Piola-Kirchhoff stress $\boldsymbol{P}$ along longitudinal (P_{zz}) and circumferential $(P_{\theta\theta})$ directions can be written as:

$$P_{zz} = 2\left[(1-d^m)c_{10}\lambda_{zz} + (1-d^f_{A_1})k_1(\lambda_{zz}^2-1)e^{k_2(\lambda_{zz}^2-1)^2}\lambda_{zz}\right] - p\frac{1}{\lambda_{zz}} \qquad \text{(S1)}$$

$$P_{\theta\theta} = 2\left[(1-d^m)c_{10}\lambda_{\theta\theta} + (1-d^f_{A_2})k_3(\lambda^2_{\theta\theta}-1)e^{k_4(\lambda^2_{\theta\theta}-1)^2}\lambda_{\theta\theta}\right] - p\frac{1}{\lambda_{\theta\theta}} \qquad \text{(S2)}$$

with Lagrangian multiplier p as:

$$p = \frac{2(1-d^m)c_{10}}{\lambda_{zz}} \qquad \text{(S3)}$$

$$p = \frac{2(1-d^m)c_{10}}{\lambda_{\theta\theta}} \qquad \text{(S4)}$$

The analytical model was fitted by using least square method in Matlab. Circumferential and longitudinal stress values and related stretch values were derived from experiments. Damage variables d^m, $d^f_{A_1}$ and $d^f_{A_2}$ are defined as in Table 1. Thus, Holzapfel material parameters c_{10}, k_1, k_2, k_3, k_4 and damage model parameters $\boldsymbol{\Xi_0^m}$, $\boldsymbol{\Xi_F^m}$, $\boldsymbol{\Xi_0^{fA_1}}$, $\boldsymbol{\Xi_F^{fA_1}}$, $\boldsymbol{\Xi_0^{fA_2}}$, $\boldsymbol{\Xi_F^{fA_2}}$ were fitted so that stress-stretch response of the analytical model followed the experimental one.

Supplementary Table S1. Clinical features of patients with peripheral artery disease (PAD) enrolled in the study: demographical (age, sex) and cardiovascular risks (hypercholesterolemia, smoking, hypertension, coronary artery disease).

Patient characteristics	
Age (mean ± SD)	72±12
Sex	Male (100%)
Hypercholesterolemia (%)	33
Smoking (%)	25
Hypertension (%)	62.5

Coronary artery disease	12.5%
Statins (%)	25

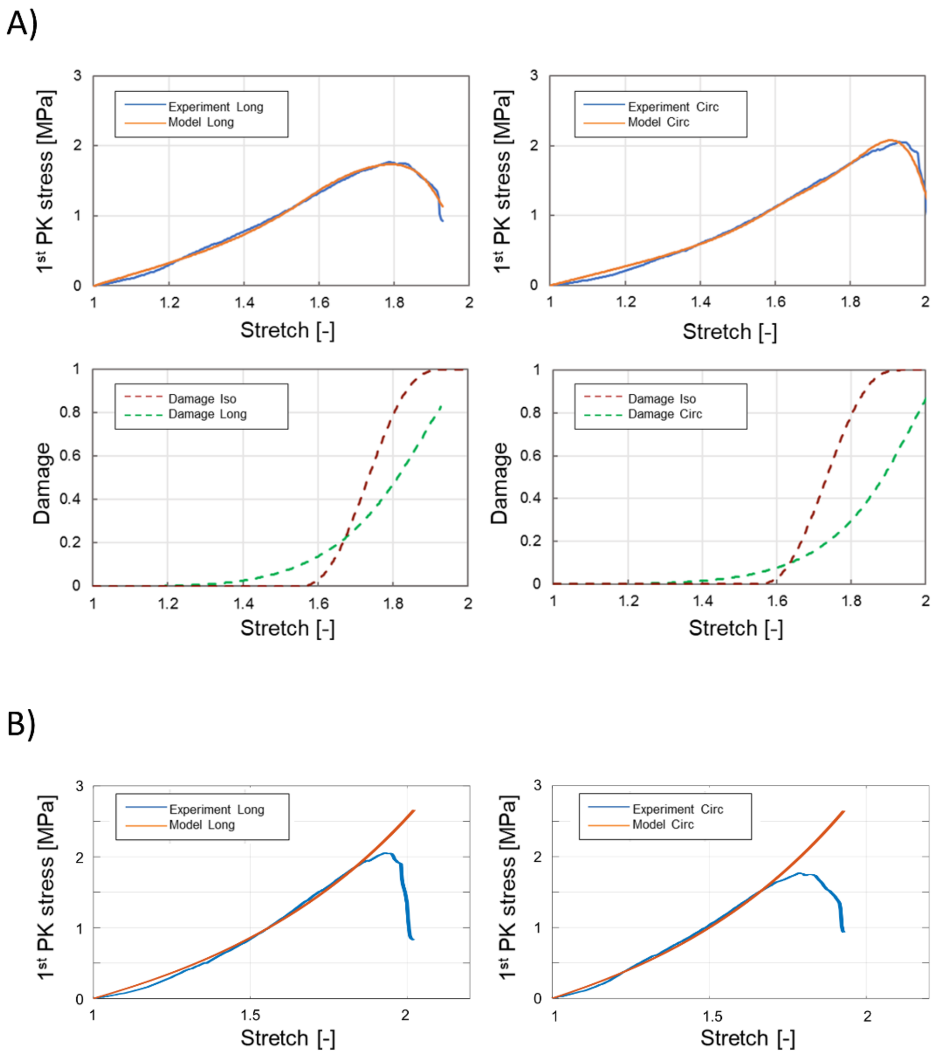

Supplementary Figure S1 A) Calibration of the analytical model for the media layer. Experimental uniaxial stress-stretch response of a human superficial femoral artery (SFA) sample along longitudinal loading (left) and circumferential loading (right), in blue, and model fitting in orange. Green and brown dashed curves represent the isotropic and longitudinal (left) and circumferential (right) damage, fitted to reproduce the experimental data. B) Calibration of the analytical model for the adventitia layer.

Experimental uniaxial stress-stretch response of a human SFA sample along longitudinal loading (left) and circumferential loading (right), in blue, and model fitting in orange

Agent-based model inputs

Damage-based input (*Dinput*) was defined as the product between the local damage computed through the PTA module and a generic post-intervention time-varying inflammatory curve [2] (Supplementary Figure S2), as introduced in a previous study of the authors [3]. This curve exhibited a peak around day 3 and vanished at 1 month after the intervention.

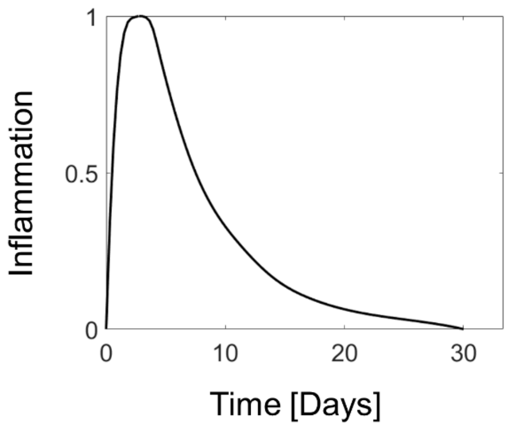

Supplementary Figure S2 Generic inflammatory curve, inspired from literature [2]

Wall shear stress (WSS) – based input (*WSSinput*) formulation reflected the evidence that an inverse relationship exists WSS and smooth muscle cell (SMC) activities, namely low WSS promotes increased SMC synthetic and proliferative activity, contributing to restenosis [4]. Thus, the overall effect of WSS on SMCs was modelled phenomenologically, without explicitly modeling endothelial cells [3,5,6].

Consequently, *WSSinput* was obtained by the combination of (i) $D(WSS)^i$, as defined in Table 5, representative of a WSS-derived level of endothelial dysfunction at the lumen wall sites of the ABM (Supplementary Figure S3) and (ii) the propagation of $D(WSS)^i$ across the thickness of the media layer using a sinusoidal function (Table 5). In particular, $D(WSS)^i$ consists in a sigmoid-shaped function (Supplementary Figure S3) that represents the level of endothelial dysfunction associated to the a WSS value. $D(WSS)^i$ is governed by the parameters L_1=-7.55 and L_2=1, defining the slope and the WSS value at which D = 0.5, respectively. Accordingly, higher levels of D are found in luminal regions exposed to low WSS. L_1 and L_2 were defined by following the methods described in [5], and considering a WSS range of [0 2] Pa. Thus, L_1 and L_2 were set to guarantee D(WSS=0)>0.9 and D(WSS=2)<0.1.

Then, within the media (at a distance *x<dist* from the *i*th site), *WSSinput* was computed as the sum of the decays of $D(WSS)^i$, described as the cosine functions, with *Amp*, amplitude of the cosine function. The value of the parameter *dist* was set equal to 15 as in previous works [3,5,6]. Differently, Amp was set to 0.14 to account for the larger intima/media area.

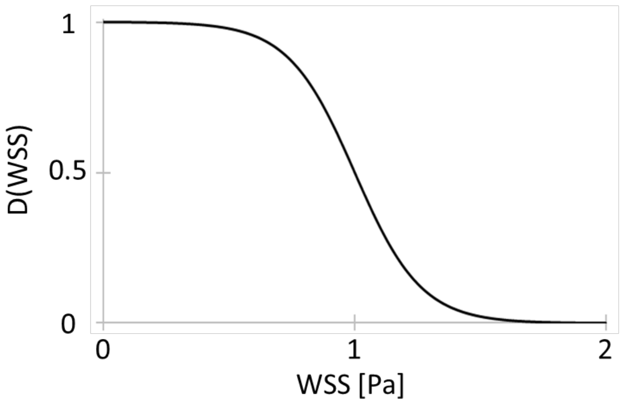

Supplementary figure S3 Representation of the variable D(WSS), accounting for the impact of the wall shear stress (WSS) on the level of endothelial dysfunction

SUPPLEMENTARY RESULTS

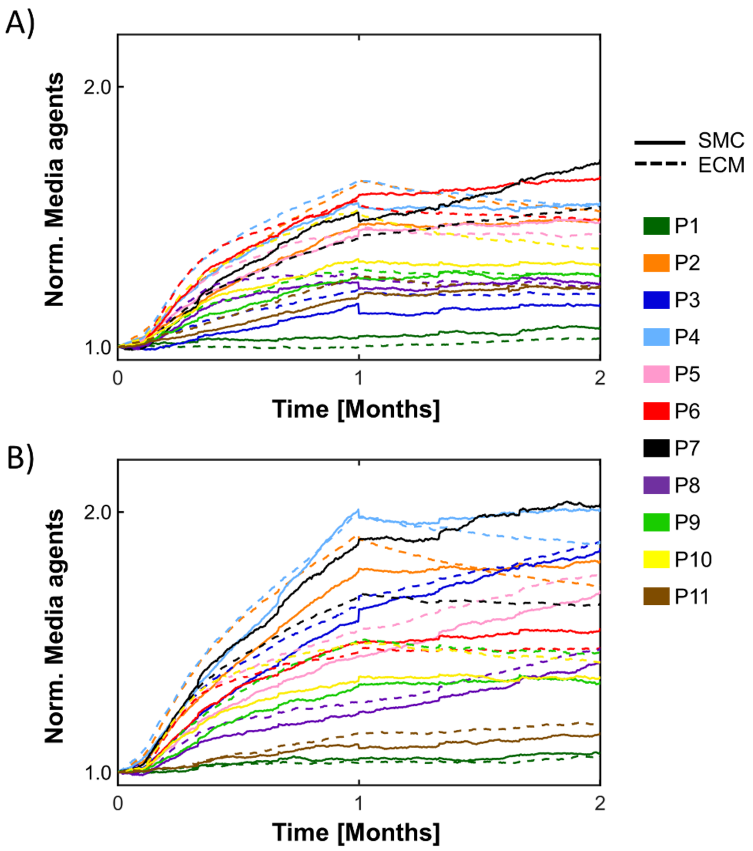

Supplementary figure S4 The temporal trends of the normalized medial content of smooth muscle cells (SMC) (solid line) and extracellular matrix (ECM) (dashed line) along 2 simulated months in the 11 agent-based model (ABM) planes of cases A and B. P1 to P11 correspond to the 11 ABM planes

SUPPLEMENTARY REFERENCES

[1] Ramella M, Bernardi P, Fusaro L, Manfredi M, Casella F, Porta CM, Nicolai L, Galeazzi E, Boldorini R, Settembrini AM, Settembrini P, Marengo E, Cannas M, Boccafoschi F, Relevance of inflammation and matrix remodeling in abdominal aortic aneurysm (AAA) and popliteal artery aneurysm (PAA) progression. Am. J. Transl. Res. 2018; 10: 3265–3275.

[2] Edelman ER, Rogers C, Pathobiologic responses to stenting. Am. J. Cardiol. 1998; 81: 4E-6E. https://doi.org/10.1016/s0002-9149(98)00189-1.

[3] Corti A, Colombo M, Migliavacca F, Berceli SA, Casarin S, Rodriguez Matas JF, Chiastra C, Multiscale agent-based modeling of restenosis after percutaneous transluminal angioplasty: Effects of tissue damage and hemodynamics on cellular activity. Comput. Biol. Med. 2022; 147: 105753. https://doi.org/10.1016/j.compbiomed.2022.105753.

[4] Koskinas KC, Chatzizisis YS, Antoniadis AP, Giannoglou GD, Role of endothelial shear stress in stent restenosis and thrombosis: Pathophysiologic mechanisms and implications for clinical translation. J. Am. Coll. Cardiol. 2012; 59: 1337–1349. https://doi.org/10.1016/j.jacc.2011.10.903.

[5] Corti A, Colombo M, Rozowsky JM, Casarin S, He Y, Carbonaro D, Migliavacca F, Rodriguez Matas JF, Berceli SA, Chiastra C, A predictive multiscale model of in-stent restenosis in femoral arteries: linking hemodynamics and gene expression with an agent-based model of cellular dynamics. J. R. Soc. Interface. 2022; 20210871. https://doi.org/https://doi.org/10.1098/rsif.2021.0871.

[6] Corti A, Migliavacca F, Berceli SA, Chiastra C, Predicting 1-year in-stent restenosis in superficial femoral arteries through multiscale computational

modelling. J. R. Soc. Interface. 2023; 20: 20220876. https://doi.org/10.1098/rsif.2022.0876.
